# Corrosion and Mechanical Micro-Interaction Behavior of Metal Materials

**DOI:** 10.3390/ma18174114

**Published:** 2025-09-01

**Authors:** Ming Liu, Ziyuan Zhao

**Affiliations:** 1 Department of Materials Science and Engineering, Xi’an University of Technology, Xi’an 710048, China; zhaoziyuan@xaut.edu.cn; 2 Shaanxi Province Key Laboratory of Corrosion and Protection, Xi’an University of Technology, Xi’an 710048, China

Corrosion is essentially a degradation process at the microscopic scale of materials, involving chemical reactions at the atomic and molecular levels, which directly affect the microstructure of materials and thereby lay the foundation for changes in mechanical behavior [1,2,3,4,5]. In electrochemical corrosion, anodic dissolution and cathodic reduction of metals in humid environments leads to local ion exchange, forming corrosion pits or crack sources [6]. The process preferentially occurs at grain boundaries, dislocation lines, or impurity sites, for these regions have irregular atomic arrangements, higher energy, and are prone to selective dissolution [7]. For example, the atomic bonding at grain boundaries is weaker in corrosion, and the lattice continuity will be disrupted after corrosive media like Cl^−^ ions invade, creating micron-sized pores or gaps [8]. These microscopic defects act as stress concentration points, significantly weakening the material’s load-bearing capacity: when external loads are applied, the stress field around defects amplifies, accelerating dislocation slip and plastic deformation, ultimately reducing yield strength and fatigue limit [9]. Experiments show that corrosion pits can greatly shorten the fatigue life of metals, and corrosion products accumulate at microscopic interfaces, increasing brittleness tendency [10]. In summary, corrosion provides microscopic “seeds” for mechanical failure by altering atomic bonding and introducing structural inhomogeneity.

Mechanical behavior at the microscopic level relies on mechanisms such as dislocation motion, grain strengthening, and phase transformation, while corrosion interferes with these processes by introducing environmental factors, leading to synergistic degradation [11,12]. The strength, toughness, and other properties of materials originate at the atomic scale: dislocation slip is the basis of plastic deformation, while grain boundaries and precipitates strengthen by hindering dislocation motion. However, corrosive environments (such as acidic media or hydrogen-containing gases) locally destroy these mechanisms [13]. For instance, in stress corrosion cracking (SCC), corrosive media promote dislocation multiplication: hydrogen atoms diffuse to dislocation cores, reducing stacking fault energy, making dislocations more prone to slip and forming micro-cracks [14]. Microscopically, this manifests as an “anodic dissolution-dislocation coupling” model: corrosion-induced pits become stress concentration sources; dislocations accumulate at pit edges, leading to crack initiation. Corrosion fatigue (CF) is more complex: corrosion products form at crack tips under cyclic loading, hindering crack closure and accelerating propagation rates [15]. Experimental data indicate that the fatigue crack growth rate of steel can increase by more than 10 times in seawater due to hydrogen atoms adsorbing at crack fronts [16]. Therefore, corrosion not only weakens microscopic strengthening mechanisms but also amplifies local defects into macroscopic failures through environmental-mechanical synergistic effects.

The interaction between corrosion and mechanical behavior at the microscopic level manifests as a dynamic feedback loop involving defect evolution, stress redistribution, and environmental response, which has profound implications for engineering applications [17,18]. In microscopic models, the formation of corrosion products interacts with mechanical loading: oxide films rupture under tensile stress, exposing fresh surfaces and accelerating corrosion; simultaneously, the volume expansion caused by corrosion induces internal stresses under constrained conditions, promoting micro-crack propagation [19]. This synergistic mechanism is particularly prominent in SCC and CF: for example, Cl^−^ ion-induced local corrosion combined with cyclic loading leads to intergranular cracks growing at rates of μm/cycle in aerospace aluminum alloys, ultimately causing sudden fracture [20,21]. From a practical perspective, this association explains numerous failure cases, such as pipeline leaks in corrosive soils or bridge fatigue failures in humid environments. Preventive strategies must start from the microscopic level: isolating corrosive media through surface treatments or enhancing grain boundary stability through alloy design. In conclusion, understanding the microscopic correlation between corrosion and mechanics aids in developing high-reliability materials.
